# Perosomus Elumbis in Piglets: Pathological, Radiological and Cytogenetic Findings

**DOI:** 10.3390/ani11041132

**Published:** 2021-04-15

**Authors:** Giuseppe Piegari, Emanuele D’Anza, Dario Costanza, Francesco Prisco, Leonardo Meomartino, Ilaria d’Aquino, Sara Albarella, Orlando Paciello, Francesca Ciotola

**Affiliations:** 1 Department of Veterinary Medicine and Animal Production, Unit of Pathology, University of Naples Federico II, Via Delpino 1, 80137 Naples, Italy; francesco.prisco@unina.it (F.P.); paciello@unina.it (O.P.); 2 Department of Veterinary Medicine and Animal Production, Unit of Genetics, University of Naples Federico II, Via Delpino 1, 80137 Naples, Italy; emanuele.danza@unina.it (E.D.); sara.albarella@unina.it (S.A.); francesca.ciotola@unina.it (F.C.); 3 Interdepartmental Centre of Veterinary Radiology, University of Naples Federico II, Via Delpino 1, 80137 Naples, Italy; dario.costanza@unina.it (D.C.); meomarti@unina.it (L.M.)

**Keywords:** perosomus elumbis, pig, congenital malformation, cytogenetic examination, pathological exam, spinal cord, CT, radiography

## Abstract

**Simple Summary:**

Perosomus elumbis (PE) is a rare congenital condition characterized by malformations localized mainly at the lumbo-sacral column. PE is reported in the literature as a description of single or very few cases. Although some authors have suggested a possible genetic etiology, the cause of PE has been poorly investigated. Here we report the first extensive pathological, radiological and cytogenetical description of eight cases of PE detected in two consecutive births of a Casertana pig sow, bred in an extensive farm located in Campania region, southern Italy. In this study, we also performed an epidemiological investigation to assess the rate of piglets born dead and malformations in the farm. The gross and radiological examination showed a broad range of malformations, such as skeletal and visceral abnormalities, but cytogenetic investigation did not show chromosome alterations. Finally, the retrospective data from the farm showed a low frequency of malformations in newborn pigs in the year of PE cases observation. These findings, taken together, suggest a specific genetic etiological basis as cause of PE in pigs but exclude chromosomal abnormalities.

**Abstract:**

Perosomus elumbis (PE) is a rare congenital condition characterized by agenesis of the lumbar, sacral and coccygeal vertebrae. Perosomus elumbis has rarely been reported in literature as morphological description of singles or few cases. Here we report the first extensive description of eight cases of PE detected in two consecutive litters from the same parents of Casertana pig breed. In August 2018, eight piglets were investigated for multiple malformations. All malformed animals, but one, died in the first day of life. The survivor piglet died at 23 days of age. Pathological, radiological and cytogenetic examination was performed. Furthermore, a farm epidemiological investigation was carried out to investigate the percentage of piglets born dead or with malformations in 2018. The radiological and pathological exams showed skeletal abnormalities at the spinal cord level and visceral malformations. Cytogenetic investigations showed a normal chromosome arrangement. Finally, epidemiological investigation revealed a low prevalence of malformations in newborn pigs, equal to 0.5% of the total birth rate of the farm. Our findings report the first extensive description of PE cases in pigs and suggest an underestimation of this malformation in veterinary medicine. Our findings also suggest a specific genetic etiological basis as cause of PE in pigs and exclude chromosomal abnormalities. Further studies will be performed to confirm this hypothesis.

## 1. Introduction

Perosomus elumbis (PE) is a rare congenital condition, incompatible with life, and characterized by malformation of the lumbar, sacral and coccygeal vertebrae [1]. This congenital defect is also associated with musculoskeletal alterations of the hind limbs, such as skeletal abnormalities and muscle atrophy [1]. Perosomus elumbis has been previously reported in a broad range of domestic animals such as cattle, sheep [2], pigs [3,4], dogs [5] and horses [6]. However, it is currently considered more common in cattle than in other species. The etiology of PE has been poorly investigated. Although some authors have suggested a possible genetic basis, no studies have investigated chromosomal or genomic alterations in the PE cases reported in the past. Karakaya et al. [7] described a case of PE in a Holstein calf infected with bovine viral diarrhoea virus (BVD), suggesting a possible role of this virus in the genesis of the malformation. Therefore, a possible microbiological etiology has been supposed in cows. Overall, scientific reports on PE are sporadic and characterized only by descriptions of single or few cases [1,2,3,4,5,6,7,8,9]. In pigs, to the best of our knowledge, only 10 PE cases have been reported [2,8,9]; no familial form of this congenital defect has also been reported both in pigs and other animals. Therefore, it is considered a rare and sporadic malformation with a reduced economic impact within animal production. However, the real incidence of congenital malformations in domestic animal farming is still underestimated [10,11]. Indeed, in many cases the malformations are not detected or not declared, and often the breeder decides to eliminate from the herd the affected subjects and the sires and dams without any veterinary consult. This causes the loss of information on the incidence of the malformations in domestic animals, and the loss of animal models of diseases useful for identifying causes, defining diagnostic techniques and possibly therapies for those conditions. 

Cytogenetic analyses, like conventional and banded karyotype, should be the first to be performed in cases of congenital malformation of unknown genetic causes [12]. They are commonly used for the evaluation of different kind of congenital malformation like disorders of the genital apparatus [13,14,15] and musculoskeletal defects [16,17,18,19]. In pigs, balanced reciprocal translocations are relatively common and occurs at an approximate rate of one in 200 boars [20]. In this species, chromosomal rearrangements of a single parent have been found to be responsible for the malformation observed in the offspring [21]. Furthermore, congenital malformations of unknown genetic causes could easily spread to other farms thanks to the extensive use of artificial insemination in sow. Indeed, Artificial insemination is strongly diffused in livestock population and allows to speed up genetic progress and obtain a copious amount of offspring from a single male breeder. However, artificial insemination has to be associated with the early identification of chromosomal and genetic anomalies of carriers in order to prevent the spreading of congenital malformations or reduced fertility in livestock populations, thus avoiding economic losses to animal farms [22]. Here we report the first extensive description of eight cases of PE detected in two consecutive litters from the same parents of Casertana pig breed. This is an autochthonous pig breed reared in Southern Italy resulting from roman pig [23], with a more stable genome than those of other Italian species and breeds, probably related to different breeding management and environmental factors [24].

Particular attention has been paid to morphological description to confirm the diagnosis and to cytogenetic examination to investigate chromosome aberrations in the etiology of this pathology. 

## 2. Material and Methods 

### 2.1. Ethical Statement

The Ethical Animal Care and Use Committee of University of Naples Federico II pre-approved all procedures used in this research study (Prot. Nr. PG/2020/0014509).

### 2.2. Case History

In August 2018, eight newborn piglets of Casertana pig, birthed by a five-year-old sow, bred in an extensive farm located in the Campania region, Southern Italy, were investigated by the Laboratory of Veterinary Genetics of the Department of Veterinary Medicine and Animal Production, University of Naples Federico II, Italy, for multiple malformations detected immediately after birth. Of the assessed newborns, five out of eight had no malformations, and three out of eight showed dysplasia of the caudal part of the spina and spinal cord and hind limbs. All malformed animals showed clinical symptoms characterized by motor ataxia of the hind limbs. Two out of the three affected piglets died in the first days of life, while one piglet survived for 23 days and subsequently died due to respiratory complications. All animals were sent to the Unit of Pathology of the Department of Veterinary Medicine of the University of Naples Federico II for further investigation. During the period of observation, the sow gave birth to nine death piglets, five of which were malformed, and four did not show macroscopically evident malformations. All malformed animals were died at the time of clinical examination and showed dysplasia of the caudal part of the spina and spinal cord and hind limbs. All piglets were sent to the Department of Veterinary Medicine of the University of Naples Federico II for subsequent necropsy, genetic and radiologic investigations. The sow died of dystocia complications at the second birth.

### 2.3. Epidemiological Investigation at the Farm

The breeder was interviewed to obtain information about the composition and number of animals’ farm and the percentage of piglets born dead and with malformations in 2018. Moreover, data about other litters from the same boar and about the relatives of the sow were sought. Data about the vaccination program of the farmed animals and the results of serological monitoring for Aujeszky’s disease, in compliance with the National Control Plan for Aujeszky’s disease in the porcine species (GU n.251 of the 26-10-1994), were asked.

### 2.4. Radiological Exams

One piglet of the first birth was submitted to in vivo radiography of the trunk, ultrasonography of the abdomen, total body CT and CT-myelography. All the other piglets underwent to radiographic and CT studies performed post-mortem. A total of 14 piglets were studied.

Radiographs were obtained using digital CR system (Agfa CR-X30, Agfa Italia, Milan, Italy). CT studies were performed using a helical 16-slice scanner (GE Lightspeed, General Electric Italia, Milan, Italy). Ultrasonography was performed with a general-purpose device equipped with high frequency microconvex and linear probes (Mylab Class C, Esaote, Firenze, Italy).

### 2.5. Gross Examination

Necropsies were performed on all malformed animals. All necropsies were carried out in the necropsy room of the Department of Veterinary Medicine and Animal Production of the University of Naples Federico II using a standard necropsy protocol [25,26].

### 2.6. Cytogenetic Examination

Whole blood was collected for karyotype analysis from 6 piglets (one affected and five healthy) of the first birth and both parents. Two types of cultures, one without (normal culture) and one with (RB-banded culture) BrdU at 20 ug/mL were set up for each animal as previously reported [27]. 5-BrdU (20 ug/mL) and H33258 (40 ug/mL) were added to RB-banded culture 5 h before harvesting. Colcemid was added to all cultures for 1 h, then the cells were subjected to a hypotonic treatment (KCl 0.5%) and to three fixations in methanol-acetic acid (3:1), the final one overnight. Three drops of cell suspension were air dried on cleaned and wet slides which were stained a day later (slides from normal cultures) or 10 days later (slides from RB-banded culture). Slides from normal cultures were stained with acridine orange (0.01% in a phosphate buffer, pH 7.0) for 10 min, washed in tap and distilled water and mounted in the same phosphate buffer. Slides from RB-banded culture were stained with H33258 (25 ug/mL) for 20’, washed in tap and distilled water, mounted with 2xSSC and UV exposed for 30’, washed in tap and distilled water, stained with acridine orange (0.01% in a phosphate buffer, pH 7.0) for 10 min, washed in tap and distilled water and mounted in the same phosphate buffer. Slides were observed about 24 h after being stained, or later (1 week). At least 100 and 15 metaphase plates for each animal were observed for aneuploidy detection, and for RB-banded karyotype, respectively. Karyotypes were arranged according to Gustavsson et al. [28]. 

## 3. Results

### 3.1. Epidemiological Investigation at the Farm

The farm consisted of 123 pigs. The percentage of malformations detected in the newborn piglets in 2018 was 0.5% and mainly concerned cryptorchid animals. The stillborn piglet rate was equal to 10.5% of the total birth rate. Apparently, the boar never sired piglets with a similar malformation when bred with other sows and it did not show a reduced number of piglets per litter. The sow gave birth only to the two litters previously described, both with the same boar, while there were no data about other relatives of the sow. The vaccination protocol of the breed included immunization against porcine parvovirus. The results of serological monitoring for Aujeszky’s disease were negative for all assessed pigs.

### 3.2. Radiological Exams 

**Figure 1:**
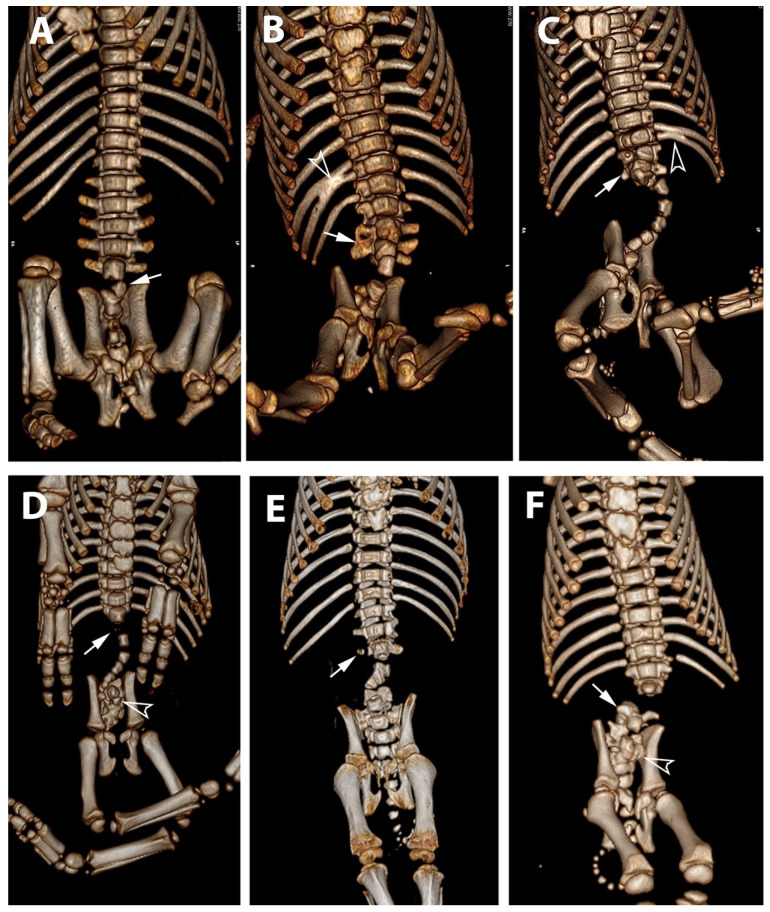
Ventral aspect of the skeleton on 3D-VR CT reconstructions with the spectrum of the malformations observed in piglets affected by Perosomus elumbis (the right side of the subjects is on the left). (**A**) Piglet #3: L6 (arrow) is present only as a bud. (**B**) Piglet #2: the lumbar tract shows a scoliotic curvature; L2-L3-L4 are irregularly fused (arrow); L5 and L6 are present as a bud; the right 14th and 15th ribs are partially fused (empty arrowhead); both the knees have an inverted angle. (**C**) Piglet #4: the lumbar tract shows a severe lordotic-scoliotic curvature; L1 is partially fused with T14 and L2 (arrow); the remnant lumbar vertebrae are present only as a bud; S1 is present as a small bud and the pelvis is tilted toward the left; there is also a partial fusion of the left 13th and 14th ribs (empty arrowhead) and a severe inversion of the left knee angle. (**D**) Piglet #5: L1 has only a slight mineralization of the body (arrow), the remnant lumbar vertebrae are present as buds with a complex lordotic-scoliotic curvature; the body of S1 is partially fragmented (empty arrowhead); the coccygeal vertebrae are absent; both the knees have an inverted angle. (**E**) Piglet #6: partial aplasia of L2 and L3 with a right fragmented lateral process (arrow); the remnant lumbar vertebrae (L4-L6) are present only as a bud. (**F**) Piglet #8: absence of the vertebrae from L2 to L4; L5 and L6 are partially fused (arrow); S1 and S2 were partially fused and irregularly fragmented (empty arrowhead).

**Figure 2:**
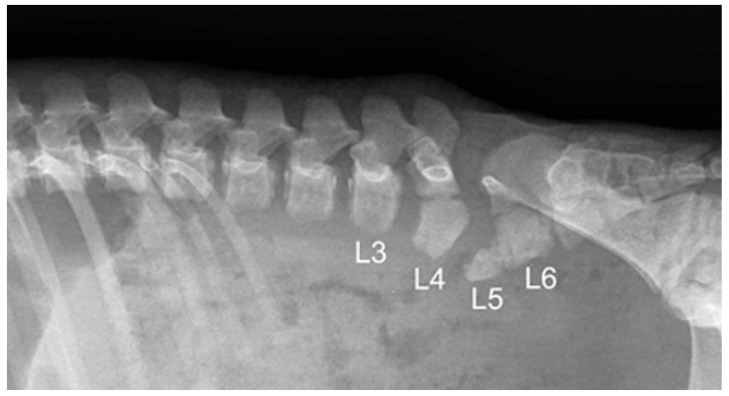
Latero-lateral radiograph of the lumbar tract of the Piglet #1. The last four lumbar vertebrae present a series of malformations: L3 miss the caudal ossification nucleus; L4 has a segmented and deformed body; L5 is present only as a “wedge-shaped” bud of the body; L6 is partially fused with L5 and with a dysplastic arch.

**Figure 3:**
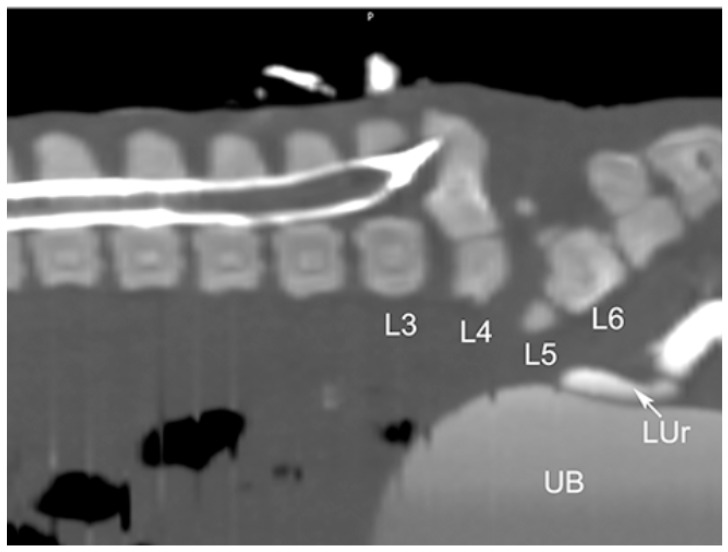
CT-myelography of the Piglet #1; sagittal multiplanar reconstruction (MPR) of the lumbar tract. The dural sac stops at the level of L3-L4 and is dorsally directed, ending in the soft tissue between a L4 bisected dorsal process (not visible in this scan plan). The lesion is compatible with myelomeniongocele and spina bifida. The affected lumbar vertebrae show the radiographic features described in Figure 2. Legend: L3-L6 = lumbar vertebrae; LUr = left ureter; UB = urinary bladder.

**Figure 4:**
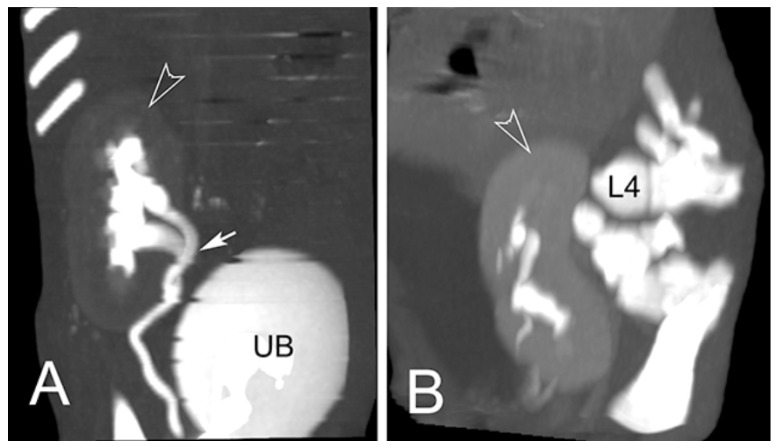
Post contrast CT of right (**A**) and left kidney (**B**) of the Piglet #1; Maximum Intensity Projection (MIP) in the dorsal plane. The kidneys (empty arrowheads) are moderately enhanced and most of the contrast medium is visible at the level of the pelvi, ureters and urinary bladder. The origin of the right ureter is duplicated (arrow). The left kidney is distorted by the compression exerted by the deformed last lumbar vertebrae. Legend: UB = urinary bladder; L4 = fourth lumbar vertebra.

**Figure 5:**
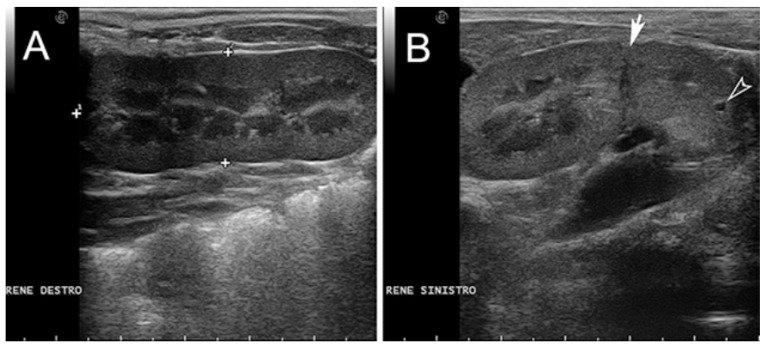
Ultrasonographic exam of the right (**A**) and left kidney (**B**) in a sagittal scan. The right kidney has an overall normal echo-structure. The left kidney has a bended shape with a deep notch at the level of the median region (arrow). At the caudal pole, it is visible a small cyst (arrowhead).

Six piglets, two of the first birth and four of second birth, did not show any radiological abnormalities. Eight piglets, three of the first birth and five of the second birth, showed multiple skeletal abnormalities of different degree of severity at the lumbo-sacral and coccygeal column characterized by total absence, partial aplasia, emivertebrae or fused and incomplete vertebrae together with the missing of the vertebral canal (Figure 1). The first piglet that also underwent the CT-myelography and abdominal ultrasonography, showed, other than the spinal malformations (Figure 2), a dorsal displacement of the last tract of the spinal cord, interrupted just caudal to L3, and with a meningo-myelocele of the last tract of the dural sac with a partial spina bifida at level of L4 (Figure 3). There was also a duplication of the most cranial tract of both ureters and caudal dislocation of the left kidney. The left kidney had a dorso-medially bended shape due to the mass effect exerted by the deformed last lumbar vertebrae (Figure 4 and Figure 5). Table 1 shows the malformations for each assessed animal.

### 3.3. Gross Examination

**Figure 6:**
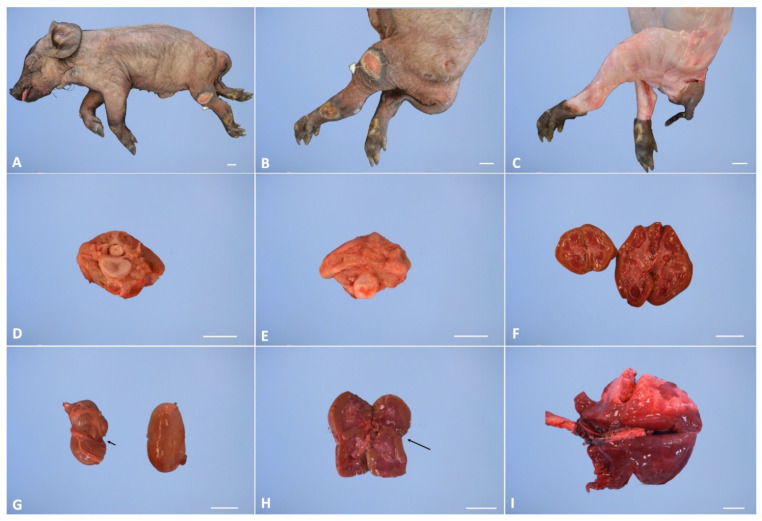
Representative pathological alterations in perosomus elumbis cases. (**A**) External appearance of perosomus elumbis, (**B**) hindlimbs dysplasia and multifocal ulcerations on hindlimbs region, (**C**) atrophy of the hind limb muscles, (**D**) lumbar vertebra shows normal development of the vertebral canal, (**E**) lumbar vertebra shows agenesis of vertebral canal, (**F**) renal hypoplasia, (**G**,**H**) renal anomalies characterized by a distinct transverse ridge on the lateral surface of the kidney running backwards (arrows), (**I**) severe and bi-lateral bronco-pneumonia. Scale bar, 2 cm.

In all malformed animals, the external examination of the cadaver showed severe atrophy of the hind limb muscles. The hindlimbs of the examined animals had dysplasia with different degrees of severity among the various examined subjects (Figure 1A–C). During inner necropsy, we observed normal development of the cervical and thoracic spinal segments; the sacro-lumbar spinal segment showed different degrees of malformation, from segmental atrophy to agenesis, in all examined cadavers. Normal development of the skull, brain and first portion of the vertebral canal was also observed. However, the lumen of the vertebral canal stopped abruptly at the level of the last lumbar vertebrae (Figure 6D,E). Inner necropsy allowed us to observe a wide range of additional visceral congenital malformations; in particular, we observed kidney hypoplasia in four out of eight cases and, in one out of eight cases, renal anomalies characterized by a distinct transverse ridge on the lateral surface of the kidney running backwards (Figure 6F,H). We also observed agenesis of adrenal glands in one out of eight cases and dislocation of the left kidney in the caudal abdomen in three out of eight cases. 

Furthermore, gross examination showed bronco-pneumonia in two out of three cases at first birth and hemorrhagic gastro-enteritis in one out of three cases (Figure 6I). Fetal pulmonary atelectasis was observed in two out of five cases of the second birth affected piglets. Table 2 shows the visceral congenital malformation and the acquired pathologies for each assessed animal.

### 3.4. Cytogenetic Examination 

Cytogenetic investigations revealed that all the analyzed animals showed a normal chromosome arrangement in accordance with their sex (2n = 38, XX or 2n = 38, XY), thus excluding that numerical or structural chromosome aberrations higher than 8 Mb are involved in the malformation found (Supplementary Figure S1).

## 4. Discussion

In this study, we reported the first extensive description of a case series of PE detected in two consecutive births from the same parents (sow and boar) of Casertana pig breed. The gross alterations of the eight cases reported in the present study appear consistent with PE and similar to those previously described in pigs and other animals of veterinary interest, such as cattle, sheep and dogs [1,2,3,4,5,6,7]. Perosomus elumbis is a congenital defect mainly characterized by musculoskeletal changes localized at the level of the spinal cord and hind limbs [1,2,3,4,5,6,7]. In the present study, radiological exams showed skeletal abnormalities of different degree of severity at the lumbo-sacral and coccygeal column, characterized by total absence, partial aplasia, emivertebrae or fused and incomplete vertebrae together with the missing of the vertebral canal. In Piglet #1 it was possible to perform the radiological exams on alive subjects and to use a CT contrast technique as well as the ultrasonography. Therefore, other than skeletal abnormalities, in that subject it was possible to assess some visceral and neurological malformations. Furthermore, the gross examination allowed us to observe severe atrophy of the hindlimb muscles. This alteration was associated with dysplasia of the hindlimbs with a different degree of severity among the examined subjects. However, the gross examination also made it possible to observe a broad range of visceral malformations located at kidney and adrenal glands level. The possibility of visceral malformations has been previously reported in the cows PE cases and could be considered a consequence of the altered development of the spine during the embryogenetic stage [1]. Indeed, kidney and adrenal glands are structures in close contact with the vault of the abdominal wall and, therefore, more subject to alterations consequent to malformations of the spinal cord. In addition, the highlighted malformations and the consequent motor ataxia could be considered an important contributing factor for the acquired pathologies observed in the piglets of the first birth, such as pneumonia and enteritis. Overall, international reports on PE are sporadic and characterized only by descriptions of single or few cases. In pigs, from 1832 until now, only 10 cases have been described [8,9]. Furthermore, no studies reported multiple cases of perosomus elumbis in consecutive births from the same parents. Therefore, this malformation is currently considered a rare and sporadic congenital disease with a reduced economic impact within animal production. Interestingly, the data presented in the present study are in apparent contradiction with the sporadic nature of the malformation, suggesting that this congenital disease could be more common in pigs than reported. Mulley et al. [29], in a study conducted on 2800 sows divided equally among four separate breeding units, showed a higher risk of antepartum and partum deaths in malformed piglets than in apparently normal piglets. Furthermore, the same experiments conducted on calves with congenital spinal syndromes suggested an underestimation of these pathologies because they were not detected during pregnancy [30,31]. Indeed, frequent are the cases in which the malformations are not detected or not declared in the farm, in this last case the breeder proceeds independently to eliminate from the herd the affected subjects and the sires and dams that generated them. In light of these observations, it is possible to suppose that the malformed piglets arriving for postpartum clinical observation could only be the “tip of the iceberg” of all affected animals; therefore, this malformation could be considered an emerging and underdiagnosed congenital disease in pigs. The etiology of PE has been poorly investigated. Although some authors have suggested a possible genetic etiology, no study has investigated genomic alterations in PE cases. In the present study, the detection of a case series of PE in two consecutive births from the same parents, could suggest an inheritance of this malformation in pigs. Furthermore, the epidemiological examination conducted on the farm allowed us to highlight a low prevalence of malformations in newborn pigs, equal to 0.5% of the total birth rate, and a percentage of dead-born piglets equal to 8.5%. These values are in line with those reported in the literature for pig breed and could suggest a low impact of environmental factors on the development of this malformation [29]. Furthermore, all farm animals were serologically negative for the Pseudorabies virus, which is commonly reported in the literature as an important cause of abortion and birth of weak piglets [32]. Therefore, although a complete virological and bacteriological panel was not performed on animals in the present study, the farm epidemiological findings and the clinic history of the exanimated subjects, taken together, suggest a specific genetic etiology for this malformation. Nevertheless, cytogenetic examination showed a normal chromosome arrangement. These findings exclude that numerical or structural chromosome aberrations higher than 8 Mb are involved in the perosomus elumbis malformation. Pig is one of the few species with a wide variability in the number of thoraco-lumbar vertebrae, with a range, in Europe animals, between 21 and 23 [33]. The development of the vertebral column is a very complex genetic process that involve a combinatorial expression of Hox genes. Previously, studies, conducted on rodents’ models, showed transformations of the vertebral column in mice with mutation of *Hox* genes [33]. In pigs, the wide variability in thoraco-lumbar vertebrae is generally attributed to the genetic selection carried out to increase body size of the animals for commercial purposes [33]. This genetic selection could be involved in the genesis of *Hox* genes mutations and perosomus elumbis malformation in pigs.

Finally, considering the data about the farm and the litters produced by both parents it is possible to hypothesize the genetic etiology of the observed malformation; however, on the basis of the analyses performed so far, it is not possible to establish whether the cause is monogenic or polygenic and whether it is totally due to the sow or linked to sow/boar genetic combination.

## 5. Conclusions

PE is commonly considered a rare congenital malformation in pigs. In the present study, we report the first extensive pathological, radiological and cytogenetical description of a case series of perosomus elumbis in pigs. The detection of a high number of cases of perosomus elumbis in two consecutive litters of the same parents suggests how this pathology could have a higher incidence than that commonly reported in literature. Finally, our findings exclude chromosomal abnormalities as causes of PE in this family of pigs but suggest a genetic etiological basis that must be investigated with specific molecular techniques. Further studies will be performed to confirm this hypothesis.

## Figures and Tables

**Table 1:** Skeletal malformations of the assessed animals. * first-birth animals ** second-birth animals.

Animal	Observed Malformations			
	Thoracic Vertebrae	Lumbar Vertebrae	Sacral Vertebrae	Coccygeal Vertebrae and Other Abnormalities
1 *	**None**	partial aplasia of L4, quite complete aplasia of the vertebral canal of L5, partial aplasia with absence of the vertebral canal of L6; scoliotic deviation toward the right.	partial aplasia of S1 with absence of the vertebral canal.	**Coccygeal vertebrae:** no visible abnormalities **other abnormalities:** duplication of the first tract of the ureters; caudal dislocation of the left kidney with a dorso-medial bended shape and some cortical small cists
2 *	**None**	the lumbar tract showed a lordotic and scoliotic curvature; L2-L3-L4 were irregularly fused and L3-L4 without a vertebral canal; L5 and L6 were present only as a bud of the body with the agenesia of the vertebral arch.	S1 and S2 missed the vertebral canal and were irregularly articulated	**Coccygeal vertebrae:** only the first five metameric were visible
3 *	**None**	the lumbo-sacral junction showed a slight lordotic curvature; L6 missed the vertebral canal.	S1 was present only as a bud of the body with the agenesia of the vertebral arch	**Coccygeal vertebrae:** Only four coccygeal vertebrae were present.
4 **	T14 was partially fused with L1.	L1 was partially fused with T14 and its body was wedge shaped (hemi vertebra) L2 had a dysplastic vertebral canal and was partially fused with L3; the residual lumbar vertebrae were present only as a bud, without a vertebral canal and with a scoliotic curvature toward the left	S1 was present as a small bud, the remnant were partially fused and with a kyphotic curvature.	**Coccygeal vertebrae:** Visible until the Co15 with some hemi vertebrae. **Other abnormalities:** Partial fusion of the left 13th and 14th ribs; the pelvis was tilted toward the left; severe hyper extension of the left knee with inversion of the normal angle.
5 **	Visible T16 with a complete aplasia of the vertebral canal.	**vertebrae** L1 had only a slight mineralization of the body and the remnant (L2-L6) were present only as a bud of the body with a complex lordotic-scoliotic curvature toward the right.	the body of S1 was partially fragmented and without the vertebral canal; S2 was visible only as a small bud; no other sacral vertebrae were visible.	**Coccygeal vertebrae:** The coccygeal vertebrae were absent. **Other abnormalities:** both the knees had an inverted angle.
6 **	Visible T15	partial aplasia of L2 with an incomplete vertebral canal; L3 without the vertebral canal and a right fragmented lateral process; the remnant lumbar vertebrae (L4-L6) were present only as a bud of the bodies and with a lordotic curvature.	partial dysplasia of the body of S1 with a not fused cranio-ventral portion of the body and without the vertebral canal.	**Coccygeal vertebrae** no visible abnormalities.
7 **	**None**	partial aplasia of L4 without the vertebral canal; L5 and L6 were present only as a small bud of the bodies.	no visible abnormalities	**Coccygeal vertebrae** no visible abnormalities **Other abnormalities** Severe hyper extension of both the knees and the tarsi.
8 **	**None**	L1 was dysplastic with bifid dorsal process and the vertebral canal communicating with the dorsal soft tissues of the lumbar tract; absence of the vertebrae from L2 to L4; L5 and L6 partially fused and with aplasia of the vertebral canal.	**Sacral vertebrae** S1 and S2 were partially fused and irregularly fragmented without the vertebral canal.	**Coccygeal vertebrae** no visible abnormalities.

**Table 2:** Visceral congenital anomalies and acquired pathologies of the assessed animals. * first-birth animals ** second-birth animals.

Animal	Sex	Visceral Congenital Anomalies	Acquired Pathologies
1 *	M	**Renal anomalies** characterized by a distinct transverse ridge on the lateral surface of the left kidney running backwards. The left kidney is dislocated in caudal abdomen **Agenesis of the left adrenal gland**	**Outer necropsy:** there are multifocal areas of ulceration over the hind limb region; these areas show an ovoid morphology, distinct edges and a diameter range from 2 × 2 cm to 5 × 3 cm; There is atrophy of the hind limb muscles and hypertrophy of the forelimbs **Inner Necropsy:** there is a severe and bi-lateral bronco-pneumonia associated with pulmonary oedema; the mediastinal lymph nodes are slightly enlarged; mild congestion is observed on both natural and cut surface
2 *	M	**Renal anomalies** characterized by hypoplasy of left kidney associated with dislocation of the left kidney in the caudal abdomen	**Outer necropsy** There is a focal sub-cutis oedema over the intermandibular region; this area shows an ovoid morphology with indistinct edges **Inner Necropsy:** there is a moderate bronco-pneumonia associated with severe pulmonary oedema; the mediastinic lymph nodes are slightly enlarged; moderate congestion is observed on both natural and cut surface
3 *	F	**Renal anomalies** characterized by hypoplasia of left kidney	**Inner necropsy:** There is a moderate haemorrhagic gastro-enteritis and sero-hemorrhagic effusion in the pericardial sac. Both liver and spleen are congested with slightly enlarged and rounded edges
4 **	F	**Pulmonary atelectasis** **Renal anomalies** characterized by hypoplasia of left kidney associated with dislocation of the left kidney in the caudal abdomen	**no macroscopic alteration**
5 **	M	**no macroscopic alteration**	**Inner necropsy:** moderate pulmonary oedema
6 **	M	**no macroscopic alteration**	**Inner necropsy:** subcutaneous haemorrhages
7 **	F	**Pulmonary atelectasis** **Renal anomalies** characterized by hypoplasia of left kidney	**no macroscopic alteration**
8 **	M	**no macroscopic alteration**	**Inner necropsy:** there is a mild sero-hemorrhagic abdominal effusion and moderate pulmonary oedema

## Data Availability

All relevant data is listed in the manuscript.

## Supplementary Materials

The following are available online at https://www.mdpi.com/article/10.3390/ani11041132/s1, Figure S1: R-banded Karyotype of (a) the affected piglet; (b) the sow and (c) the boar.
