# Supplementary figures and images for: Perosomus Elumbis in Piglets: Pathological, Radiological and Cytogenetic Findings
Source: Animals (Basel). 2021 Apr 15;11(4):1132. doi: 10.3390/ani11041132 (PMC8071472; doi:10.3390/ani11041132)

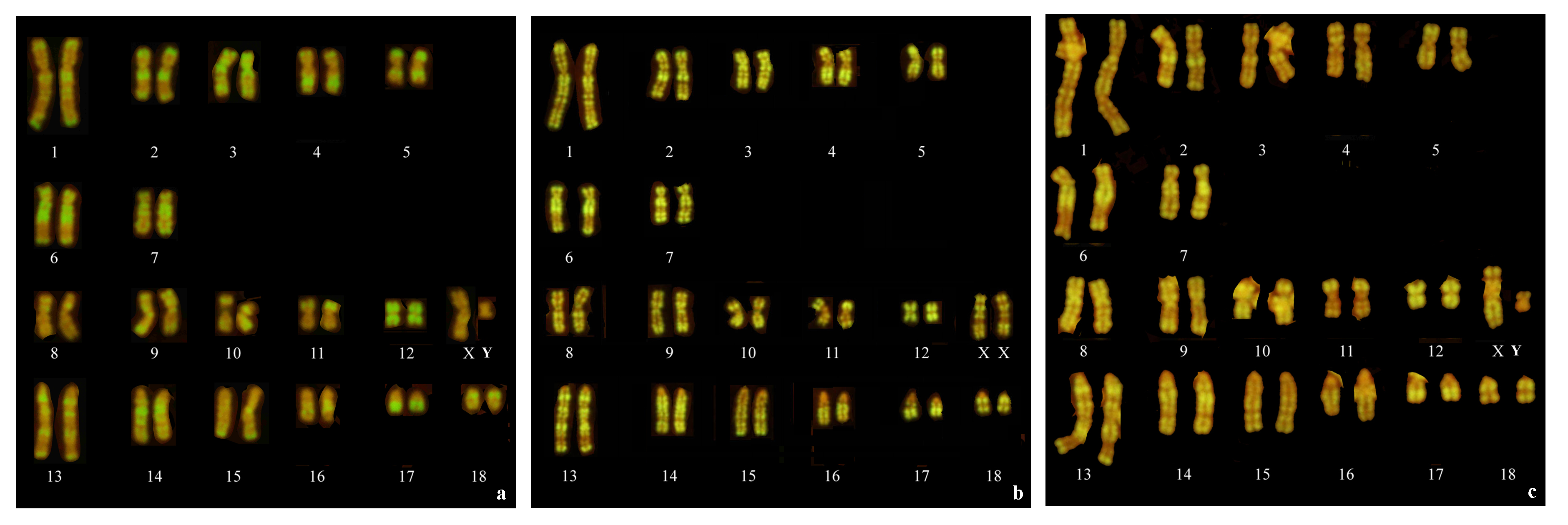

Supplement: Supplementary file 1 [file animals-11-01132-s001.zip › Suppl_Fig_PE.jpg]

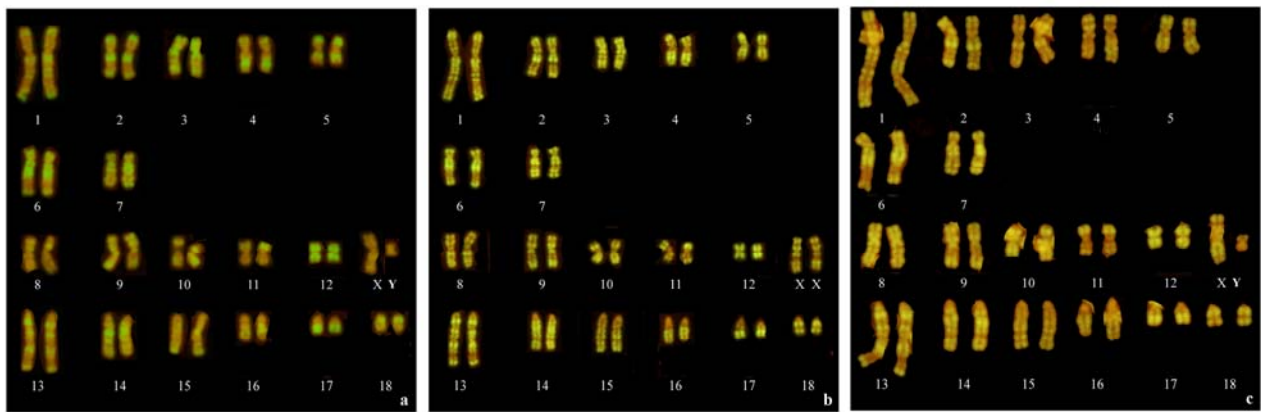

**Supplementary figure:** R-banded Karyotype of a) the affected piglet; b) the sow and c) the boar

Supplement: Supplementary file 1 [file animals-11-01132-s001.zip › Supplementary figure_PE.pdf]
